# Supplementary material for: WordCommentsAnalyzer: A windows software tool for qualitative research
Source: F1000Res. 2018 Sep 4;7:536. Originally published 2018 May 3. [Version 2] doi: 10.12688/f1000research.14819.2 (PMC6137411; doi:10.12688/f1000research.14819.2)
Supplement: Supplementary file 3 [file f1000research-7-17614-s0002.tgz › b2a79496-4ada-47c4-9682-a99ce867836d.zip!/Eight Iranian Health Journals/Journal of Health System Research _11.docx]

© Journal of Health System Research . This work is licensed under CC BY-NC 4.0 (https://creativecommons.org/licenses/by-nc/4.0/).

Volume & Issue: دوره 11، شماره 3، 1394

Title: روش‌های ارزیابی سیستم‌های رایانه‌ای تشخیصی پزشکی: مروری بر روش‌های موجود

http://hsr.mui.ac.ir/index.php/jhsr/article/view/2592

مقدمه: در حال حاضر سیستم‌های تشخیصی رایانه‌ای دارای کاربرد وسیعی در علوم پزشکی می‌باشند. این سیستم‌ها می‌توانند در تشخیص درست و به‌موقع بیماری‌ها به پزشک یاری رسانند. عملکرد این‌گونه سیستم‌ها باید به‌صورت مناسبی ارزیابی شود. در این مقاله، معیارهای ارزیابی سیستم‌های تشخیصی پزشکی مورد بررسی قرار می‌گیرد. روش‌ها: با استفاده از داده استاندارد در هر روش تشخیصی، میزان خطای سیستم بر اساس پارامترهای حساسیت، ویژگی، صحت، دقت ، سطح زیر منحنی (ROC یا receiver operating characteristic) ، F-measure،Matthews correlation coefficient  و … محاسبه شد و نقاط قوت و ضعف هر معیار مورد بررسی قرار گرفت. معیارهای ارزیابی در یک مثال پزشکی بر روی مقایسه دو روش در تشخیص عارضه قلبی بیماران، محاسبه و ارزیابی شدند. هم‌چنین ارجحیت روش‌های تشخیصی  به یکدیگر با استفاده از تست McNemar مشخص گردید. یافته‌ها: به دلیل برابر بودن نسبی تعداد افراد واقعا سالم و مریض در مجموعه داده مورد استفاده، پارامتر صحت، معیار قابل قبولی برای ارزیابی کلی دو روش بود که در دو روش به ترتیب 84 و 86٪ محاسبه شد. هر دو روش از نظر پزشکی، قابل اعتماد نبودند چون میزان خطای نوع اول بیشتر از05/0 بود. با این حال، توان تشخیصی روش دوم چون بالای 80% بود، قابل قبول می‌باشد. چون سطح زیر منحنی ROC در دو روش بین 8/0 و 9/0 بود، قدرت تشخیصی آن‌ها "بسیار خوب" می‌باشد. در نهایت، دو روش تشخیصی از نظر آماری معادل شناخته شدند. نتیجه‌گیری: ارزیابی سیستم‌های تشخیصی باید با روش‌های مناسب و معیارهای مرتبط انجام گیرد. با بکارگیری معیارهای متعدد قادر خواهیم بود توانایی روش تشخیصی را از زوایای مختلف ارزیابی نماییم.

Title: بررسی وضعیت سرطان پستان در استان کردستان با دیدگاه جغرافیای پزشکی طی سال‌های 1385 الی 1389

http://hsr.mui.ac.ir/index.php/jhsr/article/view/1521

مقدمه: سرطان یکی از بیماری‎های مزمن بشری است که فاکتورهای مختلفی در ایجاد آن نقش دارد. هدف از پژوهش حاضر بررسی توزیع جغرافیایی سرطان پستان در استان کردستان، شناخت نقاط پرخطر و عوامل جغرافیایی طبیعی و انسانی دخیل در بروز آن می‎باشد. روش‌ها: اين مطالعه از نوع توصيفي و به روش مقطعي  بوده که در آن داده‎های تحقیق با ‎استفاده‎ از نرم‎افزار آماريSPSS  تجزیه ‎و‎ تحلیل گردید و از نمودارها و آزمون‎های آماری تی و تحلیل‎ واریانس برای مقایسه استفاده‎شد و نقشه پراکندگی سرطان پستان در استان کردستان با‎ استفاده ‎از نرم‎افزار (GIS یا Geographic Information System) ورژن 9.3 طی سال‎های 1385 الی 1389 ترسیم گردید. به‎منظور بررسی علت بالا بودن نرخ مبتلایان ناشی از سرطان پستان در برخی از شهرستان‎ها، نقشه پراکندگی عناصر مس و سرب که بر روی سرطان پستان مؤثر هستند تهیه ‎شد. یافته‌ها: شهرستان‎های سنندج، سقز و مریوان به‎ترتیب با میزان بروز 59/9، 21/4 و 07/4 در هر صد‎هزار نفر در مناطق شایع بیماری قرار‎دارند. از ‎آنجا‎ که سطح معنی‌دار آماری آزمون t، 0001/0 به‎دست آمده‎است (05/0 p<)، با اطمینان 95٪ میزان بروز سرطان سینه در شهرنشین‎ها بیشتر از 4 برابر روستانشین‎ها است. مبتلایان زن (91/96٪) جامعه آماری را تشکیل داده‎اند. نتیجه‌گیری: زنان جامعه شهری گروه در‎معرض‎خطر سرطان پستان در استان کردستان می‎باشند. وجود عناصر مس و سرب در منطقه می‎توانند به‎عنوان یکی از علل بروز بیماری و پیش‎آگهی، جهت مقابله‎ با آن محسوب‎گردند. نتایج این مطالعه می‎تواند فرضیه‎هایی درباره عوامل خطر احتمالی سرطان پستان در استان کردستان ایجاد‎ کند که اثبات آن مستلزم انجام مطالعاتی مکمل از طرف مسؤولین می‎باشد.

Title: بررسی ارتباط بین نيترات آب آشاميدني و شیوع سرطان معده در استان اصفهان

http://hsr.mui.ac.ir/index.php/jhsr/article/view/1819

مقدمه: يکي از بيماري‌هاي شایعی که امروزه عامل مهم مرگ و میر بوده و از نظر جغرافياي سلامت حائز اهمیت است، سرطان معده می‌باشد. اين بيماري پراکندگي يکساني در سراسر جهان ندارد و بيشتر در مناطق جنوب شرقي آسيا ديده مي‌شود. سبک زندگی و آلودگی‌های محیطی از عوامل بروز این بیماری محسوب می‌شود. هدف از انجام این مطالعه، بررسی ارتباط بین نيترات آب آشاميدني و شیوع سرطان معده در استان اصفهان بوده است. روش‌ها: در مطالعه حاضر از داده‌های مربوط به بيماران مبتلا به سرطان معده و نيترات آب آشاميدني استفاده شده است. تهيه نقشه پهنه‌بندي اطلاعات با نرم‌افزار GIS و تحليل آماري آنها با نرم افزارهای SPSS  و EXCEL انجام شده است. یافته‌ها: کانون هاي اصلي بيماري در استان به ترتيب شهرستان‌هاي فريدن، سميرم و فريدون‌شهر مي‌باشد که مقايسه اين کانون‌ها با غلظت نيترات آب آشاميدني (مناطقي که نيترات بين 45- 20 میلی‌گرم در لیتر) نشان داد که اين مناطق بر هم مطابق هستند ( با ضريب همبستگي 42%=r و 05/0= p ). اين بيماري در مردان و عمدتاً افراد بالاي 75 سال شيوع بيشتري داشته است. نتیجه‌گیری: با توجه به نتايج به دست آمده، ارتباط معنی‌داری بین غلظت نيترات آب آشاميدني و شیوع سرطان معده در مناطق مورد مطالعه وجود داشته است.

Title: بررسی شاخص هاي بیماریابی در برنامه ادغام بهداشت روان در سیستم شبکه های بهداشتی استان اصفهان1380الی1385 ومقایسه آن با استانداردهای کشوری

http://hsr.mui.ac.ir/index.php/jhsr/article/view/1845

مقدمه: هدف از برنامه كشوري بهداشت روان رسيدن به حداقل خدمات رواني براي همه مردم كشورمان تا سال 2000 بود. در سال 1367 طرح ادغام بهداشت روان درمراقبت‌هاي بهداشتي اوليه در شهركرد و شهرضا كه هدف آن بررسي اثربخشي ادغام بهداشت روان درنظام مراقبت‌هاي بهداشتي اوليه بود، به مرحله اجرا گذاشته شد. چون نتايج اين بررسي، اثربخشي آن را نشان داد، درسال 1369 بهداشت روان به عنوان جزءنهم PHC اعلام گرديد و به تدريج دركل كشورگسترش يافت. هدف این مطالعه بررسی شاخص‌های بیماریابی این برنامه از سال 85-80 و مقایسه آن‌ها با حد انتظار کشوری بود. روش‌ها: مطالعه از نوع توصيفي مقطعي طولی بود. نمونه‌گیری به روش سرشماری وحجم نمونه شامل اطلاعات کلیه بیمارانی بود که طی سال‌های 85-80 تحت پوشش برنامه بهداشت روان قرار داشتند. این اطلاعات از طريق بررسي مستندات فرم استاندارد جمع‌آوری اطلاعات بیماران بهداشت روان کشوری وزارت متبوع صورت پذیرفت. در پايان هرسال نيز اطلاعات بيماران بهداشت روان همان سال جمع‌بندي گردید و نتایج با نرم‌افزار آماری SPSS مورد تجزیه و تحلیل قرار گرفت. دراين مطالعه بيماران شناسايي شده و تحت پوشش بهداشت روان (رواني شديد، رواني خفيف ، صرع ، عقب ماندگي ذهني و ساير موارد) در هر سال، مشخص و با حد انتظار كشوري مقايسه شدند. 05/0>P-value به عنوان سطح معنی‌داری در نظر گرفته شد. یافته‌ها: وضعیت شاخص‌های بیماریابی روانی شدید از 248/1 به 46/2 درهزار، روانی خفیف از 96/9 به 5/17 درهزار، صرع از 15/3 به26/5 درهزار، عقب مانده ذهنی از 82/3 به86/5 درهزار و سایر اختلالات روانی عصبی از 79/2 به 28/4 درهزار درطی سال های 1380تا 1385 افزایش یافت که نسبت به حد مورد انتظار کشوری بالاتر بود. نتیجه‌گیری: یافته‌های این پژوهش نشان داد که فراوانی بیماری خفیف روانی هم ازحد انتظار کشوری و هم نسبت به بیماری‌های شدید روانی، صرع، عقب‌ماندگی ذهنی و سایر اختلالات روانی عصبی بالاتر بود و لذا آموزش بیشتر بهورزان، افزایش سطح آگاهی مردم نسبت به علائم بیمارهای روانی و لزوم مراجعه به موقع به مراکز درمانی احساس می‌شود.

Title: بررسي پيشگويي‌كننده‌هاي مراقبت‌هاي بهداشت دهان و دندان در مادران باردار بر اساس تئوری رفتار برنامه‌ریزی شده

http://hsr.mui.ac.ir/index.php/jhsr/article/view/1858

مقدمه: ارتباط بیماري‌هاي دهان و دندان با عواقب نامطلوب بارداري ضرورت توجه بیشتر به وضعیت بهداشت دهان و دندان در زنان باردار را نشان می‌دهد.  تئوری رفتار برنامه‌ریزی شده به عوامل اجتماعی در ایجاد رفتار توجه خاص نشان می‌دهد. پژوهش حاضر با هدف تعيين پيشگويي‌كننده‌هاي مراقبت‌هاي بهداشت دهان و دندان در مادران باردار شهر آشخانه بر اساس تئوری رفتار برنامه‌ریزی شده در سال 1392 انجام شده است. روش‌ها: در اين مطالعه تحليلي- مقطعي، 150 نفر از مادران باردار شهر آشخانه با روش نمونه‌گيري آسان از مرکز بهداشتي درماني شهری آشخانه انتخاب شدند. اطلاعات مربوط به عوامل تئوری رفتار برنامه‌ریزی شده در خصوص مراقبت‌هاي بهداشت دهان و دندان از طريق پرسش‌نامه گردآوری شده و با استفاده از آمار توصیفی و تحلیلی، به کمک نرم‌افزارspss 18  در سطح معنی‌دار 05/0 تحليل شد. یافته‌ها: نتایج نشان داد که میانگین سنی مادران باردار مورد مطالعه 9/4  4/24 است و میانگین شاخصDMF  مادران 2/2 16/5 بود. در این پژوهش همبستگی میان قصد رفتاری با سازه‌های تئوری رفتار برنامه‌ریزی شده مورد بررسی قرار گرفت.میان قصد رفتاری با آگاهی (403/0r=) ، با نگرش (369/0 r=)، با هنجارهای انتزاعی(415/0r=)، با کنترل رفتار درک شده (488/0r=) و با رفتار (399/0r=) ارتباط مستقیم و مثبتی مشاهده شد. نتیجه‌گیری: مطالعه حاضر كارايي تئوری رفتار برنامه‌ریزی شده را در پيشگويي اتخاذ مراقبت‌هاي بهداشت دهان و دندان در مادران باردار نشان داد. از اين رو اين تئوری مي‌تواند به عنوان چارچوبي جهت طراحي و اجراي مداخلات آموزشي جهت كاهش پوسيدگي دندان استفاده شود و در دوران بارداري به حفظ سلامت مادر و كودك كمك نمايد.

Title: طرحواره های ناسازگار اولیه در بیماران مبتلا به اختلالات شخصیت مرزی

http://hsr.mui.ac.ir/index.php/jhsr/article/view/1873

مقدمه: مطالعه حاضر با هدف تعیین طرحواره‌های غالب و مقایسه‌ی طرحواره‌های ناسازگار اولیه در مبتلایان به اختلال شخصیت مرزی و افراد بهنجار انجام شده است. روش‌ها: در این مطالعه مقطعی، از میان بیماران مبتلا به اختلالات شخصیت مرزی و افراد بهنجار شهر اردبیل، 90 نفر به صورت تصادفی ساده انتخاب شدند (45 نفر در هر گروه). برای جمع‌آوری داده‌ها از فرم کوتاه پرسش‌نامه‌ی طرحواره‌ی یانگ و پرسش‌نامه چند محوری بالینی میلون استفاده شد. به منظور تجزیه و تحلیل داده‌ها از نرم‌افزار SPSS (Ver 17) و آزمون‌های آماری کای دو و تی مستقل استفاده شد. یافته‌ها: نتایج پژوهش نشان داد که بین طرحواره‌های ناسازگار اولیه در بیماران مبتلا به اختلالات شخصیت مرزی و افراد بهنجار تفاوت معنی‌داری وجود دارد. علاوه بر آن تفاوت معنی‌داری در طرحواره‌های حوزه بریدگی و طرد، خودگردانی و عملکرد مختل، محدودیت‌های مختل، گوش به زنگی بیش از حد و بازداری (001/0>P) در دو گروه مورد مطالعه دیده می‌شود. نتیجه‌گیری: بر اساس نتایج به دست آمده طرحواره‌های ناسازگار اولیه در بیماران مبتلا به اختلال شخصیت مرزی ناکارآمد می‌باشند. بر این اساس با شناسايي طرحواره هاي فوق پيش از ابتلاي فرد به اختلال شخصيت مرزي و اندازه‌گيري شدت اين طرحواره‌ها و مشخص کردن الگوي طرحواره مي‌توان راهبردهاي مداخله‌اي مناسبي را در جهت کاهش اين طرحواره به عمل آورد.

Title: ارتباط بین مصرف غذاهای ادویه دار و سوءهاضمه عملکردی در بزرگسالان ایرانی

http://hsr.mui.ac.ir/index.php/jhsr/article/view/1905

مقدمه: این مطالعه مقطعی به ارزیابی ارتباط بین مصرف غذاهای ادویه‌دار و سوءهاضمه عملکردی در گروهی از بزرگسالان ایرانی پرداخته است. روش‌ها: در این مطالعه 4763 فرد بزرگسال مورد بررسی قرار گرفتند. برای ارزیابی مصرف غذاهای ادویه‌دار، از یک پرسش‌نامه ثبت عادات غذایی استفاده شد. هم‌چنین برای ارزیابی مشکلات گوارشی از پرسش‌نامه‌ی اصلاح شده Rome III که اعتبار آن قبلا مورد سنجش قرار گرفته بود، استفاده شد. یافته‌ها: مصرف مکرر غذاهای ادویه‌دار با شانس بیشتر ابتلا به سوءهاضمه عملکردی مرتبط بود (OR: 1.64; 95% CI: 1.09-2.49). این ارتباط حتی پس از تعدیل عادات غذایی و شاخص توده‌ی بدنی معنی‌دار بود (OR: 1.66; 95% CI: 1.00-2.78). ارتباط مثبت معنی‌داری بین مصرف غذاهای ادویه‌دار با سنگینی پس از صرف غذا (OR: 1.76; 95% CI: 1.29-2.40)  و سوزش سر دل (OR: 1.78; 95% CI: 1.30-2.44) دیده شد. ارتباط معنی‌داری بین مصرف مکرر غذاهای ادویه‌دار و سیری زودرس مشاهده نشد. نتیجه‌گیری: مصرف بالای غذاهای ادویه‌دار ارتباط مستقیم معنی‌داری با ابتلا به سوءهاضمه عملکردی، سنگینی پس ازصرف غذا وسوزش سردل داشت.

Title: بررسی احساس خوب داشتن در بیماران مبتلا به دیابت نوع2 مراجعه‌کننده به واحدهای دیابت تحت پوشش مراکز بهداشتي درماني دانشگاه علوم پزشکی تهران

http://hsr.mui.ac.ir/index.php/jhsr/article/view/1978

م مقدمه: با توجه به اهمیت شناسایی ابعاد "احساس خوب داشتن (wellness) با دیابت، این مطالعه با هدف شناخت عوامل مؤثر بر ابعاد احساس خوب داشتن در بیماران دیابتی نوع 2 انجام شد. روش‌ها: اين مطالعه توصيفي- تحليلي بر روي 621 بیمار دیابتی نوع 2 به روش نمونه‌گيري تصادفي ساده انجام شد. ابزار جمع‌آوری اطلاعات پرسش‌نامه دو قسمتی بود. قسمت اول شامل سؤالات دموگرافیک و قسمت دوم ،پرسش‌نامه استاندارد سنجش احساس خوب داشتن با دیابت بود. این پرسش‌نامه پس از ترجمه و باز ترجمه وتأييد اعتبار و پایایی مورد استفاده قرارگرفت. به منظور تجزیه و تحلیل داده‌ها از نرم‌افزار SPSS نسخه 5/11 استفاده شد و نتایج در سطح کمتر از05/0معنی‌دار تلقی گرديد. یافته‌ها: میانگین نمره كلي احساس خوب داشتن 11±6/137 بود. بین جنسیت و بعد معنوی احساس خوب داشتن، بین سابقه خانوادگی و میانگین کلی احساس خوب داشتن، بین میزان تحصیلات و دوبعد فیزیکی و ذهنی، بین نوع درمان و میانگین کلی و ذهنی، بین میزان درآمد باسه بعد روانی، اجتماعی و ذهنی، ارتباط آماری معنی‌دار یافت شد. بین بعد معنوی و ذهنی با احساس خوب داشتن کل همبستگی مثبت معنی‌دار وجود داشت. نتیجه‌گیری: فاکتورهای جنسیت، سابقه خانوادگی، میزان تحصیلات، نوع درمان ومیزان درآمد از عوامل مؤثر بر احساس خوب داشتن در بیماران دیابتی نوع 2، می‌باشد.

Title: بررسی فرسودگی شغلی بهورزان شاغل در خانه های بهداشت مرکز بهداشت شماره یک اصفهان در سال 1392

http://hsr.mui.ac.ir/index.php/jhsr/article/view/1985

مقدمه: فرسودگی شغلی سندرمی روان شناختی مشتمل برسه محور خستگی هیجانی، مسخ شخصیت و احساس عدم کفایت شخصی می‌باشد، بهورزان به عنوان مراقبین سطح اول خدمات بهداشتی (Primary Health Care- PHC) در معرض انواع مشکلات جسمی و روانی می‌باشند، تحقیق حاضر با هدف "تعیین وضعیت فرسودگی شغلی در بهورزان شاغل در خانه‌های بهداشت مرکز بهداشت شماره یک اصفهان در سال 1392"جهت ارائه راهکار مناسب انجام گردید. روش‌ها: این پژوهش یک مطالعه توصيفی-تحلیلی از نوع مقطعی است که بر روی تمامی بهورزان شاغل مرکز بهداشت شماره یک اصفهان (120نفر) در سال 1392 انجام گرفت، ابزار گردآوري داده‌ها پرسش‌نامه‌ای شامل اطلاعات فردی، اطلاعات حرفه‌ای، و پرسش‌نامه مسلچ (Maslach) بود. پرسش‌نامه توسط بهورزان تکمیل و داده‌ها با استفاده از نرم‌افزار آماریSPSS  نسخه 20 و آزمون‌های آماری  ANOVA، ضریب همبستگی اسپیرمن، همبستگی پیرسون و آزمون تی مستقل تحلیل گردید. یافته‌ها: نمره کلی فرسودگی شغلی در حد متوسط با میانگین (1/42) و انحراف معیار (7/14) بود. بين نمره فرسودگي شغلي در حيطه عاطفي و حيطه مسخ شخصيت (654/0 = r و 001/0 < P) و هم‌چنین حیطه عاطفی و حیطه کاهش موفقیت فردی (572/0 = r و 001/0 < P)و حیطه مسخ شخصیت وکاهش موفقیت فردی (693/0 = r و 001/0 < P) رابطه مستقيم وجود داشت. ميانگين نمره فرسودگي شغلي كلي، بعد عاطفي و كاهش موفقيت فردي در زنان به طور معنی‌داري بيشتر از مردان بود (05/0 < P). سطح تحصيلات بهورزان با نمره فرسودگي شغلي در كل و در ابعاد مختلف رابطه معكوس داشت. با دور شدن محل سکونت بهورزان از خانه بهداشت فرسودگی شغلی در بعد عاطفی افزایش یافته است (04/=p). نتیجه‌گیری: این مطالعه نشان داد که فراوانی و شدت فرسودگی شغلی در بهورزان مرکز بهداشت شماره یک اصفهان در حد متوسط بوده و از این میان، میزان فرسودگی شغلی در زنان بیشتر از مردان بوده است که از دلایل آن می‌توان به انجام همزمان وظایف شغل بهورزی و وظایف خانه و فرزندان که باعث افزایش میزان اضطراب و استرس شده وهم‌چنین بعد مسافت وعدم بهره‌مندی از تسهیلات ایاب و ذهاب برای تردد به محل کار اشاره نمود.

Title: طراحی ابزارهای صداسنجی در بهداشت حرفه ای با استفاده از نرم افزار لب ویو (Labview)

http://hsr.mui.ac.ir/index.php/jhsr/article/view/2006

مقدمه: یکی از عوامل زیان آور در بهداشت حرفه‌ای، مواجهه با صدا و ارتعاش می‌باشد. کنترل بهتر این عامل، مستلزم‌نرم افزاری است که با کنترل لحظه به لحظه از افزایش مخاطرات جلوگیری کند. لذا این پژوهش با هدف راه‌اندازی چنین سیستمی توسط نرم‌افزار labview (لب ویو) به منظور پایش لحظه‌ای صدا انجام گردید. روش‌ها: این مطالعه از نوع تجربی است که توسط نرم افزار لب ویو، میکروفون و کارت تحلیل صدا انجام شد. اطلاعات به صورت فشار صدا از طریق میکروفون جمع‌آوری گردید و توسط کارت تحلیل صدا پردازش شد. سپس نتایج به صورت گراف یا پلات نمایش داده شد. یافته‌ها: این نرم‌افزار شامل پانل جلو و پانل عقب می‌باشد که از این طریق می‌توان اطلاعاتی مانند شبکه توزین فرکانسی و سرعت پاسخ نرم‌افزار را مشخص نمود. پس از ورود اطلاعات، اندازه‌گیری دامنه و تراز فشار صوت، آنالیز طیفی، تراکم توان صوت و سایر مشخصات صدا امکان‌پذیر بود. نتیجه‌گیری: از قابلیت‌های برنامه labview در بخش صدا می‌توان به ‌اندازه‌گیری، آنالیز فرکانس و کنترل صوت اشاره نمود که در واقع مانند تراز سنج صوت یا آنالیزور صدا عمل می‌نماید. با توجه به قابلیت‌های ذکر شده، می‌توان از این نرم‌افزار در جهت آنالیز و پردازش صدا و ارتعاش به صورت یک سیستم مانیتورینگ استفاده نمود.

Title: بررسی میزان مواجهه دندان‌پزشکان با سر و صدا در بخش‌های مختلف یکی از مراکز بزرگ دندان‌پزشکی تهران

http://hsr.mui.ac.ir/index.php/jhsr/article/view/2043

مقدمه: یکی از پیامدهای مهم انقلاب صنعتی در جهان، افزایش سطح شدت سر و صدای محیط کار است. این مقاله با هدف تبیین نقش و اهمیت میزان سطح سرو صدا در محیط کار دندان‌پزشکی، به بررسی میزان سر و صدای هندپیس یونیت‌ها و تجهیزات بخش‌های مختلف یکی از مراکز بزرگ دندان‌پزشکی شهر تهران پرداخته است. روش‌ها: در این مطالعه توصیفی - مقطعی، تراز فشار صوتی در بخش‌های ترمیم، تشخیص، اطفال، پریو، جراحی فک، اندو و بخش CSR (Central Sterile Room) با استفاده از استاندارد 3744ISO و دستگاه صداسنج B Z K مدل 2237 در ناحیه شنوایی دندان‌پزشکان اندازه‌گیری شد. یافته‌ها: نتایج مطالعه نشان می‌دهد که بالاترین میزان تراز فشار صوتی بین کلیه بخش‌های مورد مطالعه، در هندپیس آنگل یونیت‌های بخش اطفال مرکز دندان‌پزشکی بود که  dB86 شبکه A  به دست آمد. مقایسه تراز کلی فشار صوتی (LPT) در دستگاه‌های دندان‌پزشکی نشان داد که بیشترین میزان به دستگاه التراسونیک تعلق داشته که dB 8/96 شبکه A به دست آمد. نتیجه‌گیری: نتایج پژوهش حاضر نشان داد که میانگین میزان سروصداي این مرکز بالا بوده و به عنوان یک عامل افت شنوایی کارکنان در معرض می باشد. در راستای جلوگیری از مشکلات ناشی از سر و صدا مانند افت شنوایی، اجرای درست و دقیق برنامه حفاظت شنوایی و استفاده از تجهیزات حفاظت فردی مناسب مورد نیاز می‌باشد.

Title: بررسی عوامل مرتبط با مصرف روزانه میوه در دانش‌آموزان پسر ابتدایی بر اساس تئوری رفتار برنامه‌ریزی شده

http://hsr.mui.ac.ir/index.php/jhsr/article/view/2091

مقدمه: : مصرف ناکافی میوه در دانش‌آموزان یک عامل خطر برای برخی از بیماری‌های مزمن مثل سرطان‌ها و بیماری‌های قلبی عروقی در بزرگسالی محسوب می‌شود. این مطالعه با هدف بررسی عوامل مرتبط با مصرف روزانه میوه در دانش‌آموزان پسر شهرستان چالدران انجام شده است. روش‌ها: در این مطالعه مقطعی 184 دانش آموز ابتدایی به طور تصادفی از مدارس پسرانه انتخاب شدند و پرسش‌نامه مربوط به اطلاعات فردی، سازه های تئوری رفتار برنامه‌ریزی شده و وضعیت مصرف روزانه میوه را تکمیل کردند. اطلاعات پس از جمع‌آوری با SPSS نسخه 20 و آنالیزهای آماری همبستگی پیرسون و تحلیل رگرسیون خطی مورد تجزیه و تحلیل قرار گرفت. یافته‌ها: نتایج نشان داد که1/32% دانش‌آموزان 1 بار ،2/58% 2 بار ، 2/9% 3 بار و فقط 5/0% آن‌ها 4 بار در روز میوه مصرف می‌کنند. براساس آنالیز رگرسیون خطی، نگرش و هنجارهای انتزاعی قدرت پیش‌بینی‌کننده بیشتری برای قصد مصرف میوه (001/0P<،36F=، 19/0=) و هنجارهای انتزاعی و قصد رفتاری دارای قدرت پیش‌بینی کنندگی بیشتری برای مصرف میوه در دانش آموزان بودند (001/0P<،36F=، 36/0=). نتیجه‌گیری: مصرف میوه در دانش‌آموزان کمتر از مقدار توصیه شده سازمان بهداشت جهانی است. هم‌چنین براساس یافته‌های این مطالعه بایستی در طراحی مداخلات آموزشی برای دانش آموزان ابتدایی بر متغیرهای قصدرفتاری و هنجارهای انتزاعی توجه بیشتری کرد.

Title: مقایسه کارایی فرآیند انعقاد الکتریکی و انعقاد الکتریکی –شناورسازی در حذف رنگ راکتیو بلک 5 در محلول‌های آبی

http://hsr.mui.ac.ir/index.php/jhsr/article/view/2110

مقدمه: رنگزاهای مصنوعی از جمله آلاینده‌های زیست محیطی هستند که مصرف آن‌ها روز به روز در حال افزایش است. در این پژوهش تصفیه‌پذیری پساب‌های آلوده به رنگ راکتیو بلک 5 به روش انعقاد الکتریکی و انعقاد الکتریکی- شناورسازی در یک راکتور در مقیاس آزمایشگاهی با استفاده از الکترود آلومینیوم مورد مطالعه قرار گرفت. روش‌ها: در این مطالعه، یک راکتور الکتروکواگولاسیون مجهز به سیستم شناورسازی با هوای محلول در مقیاس آزمایشگاهی با حجم تقریبی 1 لیتر مجهز به 4 الکترود از جنس آلومینیوم با ابعاد 200*20*2 میلی متر مورد استفاده قرار گرفت. تأثیر پارامترهای بهره‌برداری نظیر pH محیط، شدت دانسیته جریان، زمان تماس، غلظت اولیه رنگ و شناورسازی مورد بررسی قرار گرفت یافته‌ها: نتایج نشان داد که بیشترین کارایی فرایند انعقاد الکتریکی در حذف رنگ به میزان 81% در محیط خنثی از نظر pH، دانسیته جریان mA/cm271/12 و غلظت رنگ mg/L 100 حاصل شد. نتایج دیگر این مطالعه حاکی از تأثیرگذاری بیشتر فرایند توام انعقاد الکتریکی شناورسازی در مقایسه با فرایند انعقاد الکتریکی در حذف رنگ در شرایط بهینه بهره‌برداری بود. .نتیجه‌گیری: : فرآیند انعقاد الکتریکی و انعقاد الکتریکی- شناورسازی با کاربرد الکترودهای آلومینیوم می‌تواند فرایندی امیدوارکننده برای حذف رنگ باشد.

Title: انتخاب راهبرد مناسب حفاظت از سرمایه های سازمانی در مدیریت بحران با رویکرد تلفیقی SWOT و AHP

http://hsr.mui.ac.ir/index.php/jhsr/article/view/2235

مقدمه: عليرغم طرحريزي جهت فعاليت‌هاي عادي، بسياري از سازمان‌ها فاقد طرحريزي نظامند مديريت شرايط اضطراري هستند. سازمان‌ها بايد براي بحران‌ها داراي اهداف و راهبردهاي از پيش تعريف شده باشند. با تعريف اهداف و راهبردهاي شرايط اضطراري، بسياري از ابهامات پاسخ برطرف شده و مسؤوليت‌ها، هدايت و رهبري به خوبي انجام خواهند شد. روش‌ها: با استفاده از روش دلفي، فهرستي از نقاط ضعف و قوت دروني و فرصت‌ها و تهديدات بيروني در سيستم‌هاي پاسخ اضطراري تهيه شد. سپس براي نقاط ضعف و قوت دروني و فرصت‌ها و تهديدات بيروني، آناليز (SWOT یا strengths, weaknesses, opportunities, and threats) انجام شد و راهبردهاي مناسب استخراج شد. راهبردها در سه گزينه خلاصه شدند و در مرحله بعد با استفاده از روش (AHP یا Analytic Hierarchy Process) ، گزينه ارجح انتخاب شد. یافته‌ها: براي سازمان مورد مطالعه، راهبرد تطبيقي مناسب‌ترين راهبرد مديريت بحران بود. راهبرد پاسخ اشتراكي در شرايط اضطراري به عنوان راهبرد تطبيقي در نظر گرفته شد. در اين راهبرد سعي مي‌شود با رفع نقاط ضعف داخلي، از فرصت‌هاي موجود خارجي استفاده شود. نتیجه‌گیری: همانند شرايط عادي سازمان، بايد اهداف و راهبردهاي سازمان در شرايط اضطراري تعريف و مدون شوند. بهتر است اين استراتژي‌ها با روش‌هاي ساختارمند انتخاب شده و به صورت مداوم به روز شوند.

Title: ارزیابی مواجهه با رنگ تارتارازین به روش احتمالی از طریق مصرف شیرینی های سنتی ( نبات و پولکی ) در استان اصفهان.

http://hsr.mui.ac.ir/index.php/jhsr/article/view/2261

مقدمه: گزارش‌های پراکنده در کشور مبنی بر تقلب در استفاده از رنگ مصنوعی تارتارازین به جای زعفران در آب نبات‌های سنتی (نبات و پولکی) ضرورت انجام مطالعه ارزیابی خطر دریافت تارتارازین از طریق مصرف این دو شیرینی را ایجاب می‌کند. لذا پژوهش حاضر با هدف ازریابی خطر دریافت این رنگ، طی یک مطالعه توصیفی، میزان مواجهه با تارتارازین را از طریق نقشه مصرف این دو شیرینی در استان اصفهان و هم‌چنین حضور و غلظت آلودگی نمونه‌های تهیه شده از این استان را تعیین نمود. روش‌ها: در این مطالعه تعداد 500 پرسش‌نامه از طریق مدارس استان در اختیار خانواده‌های مرکز استان و تعدادی از شهرستان‌ها  قرار گرفت و اطلاعاتی در مورد وضعیت دموگرافیکی و میزان مصرف نبات و پولکی جمع‌آوری شد. برای بررسی میزان رنگ تارتارازین. تعداد 60 نمونه شامل 35نمونه نبات و 25 نمونه پولکی طبق دستور‌العمل استاندارد 740 با روش کروماتوگرافی لایه نازک (TLC یاTin Layer Chromatography) مورد آزمایش قرارگرفت. ازروش اسپکتروفتومتری نیز جهت اندازه‌گیری کمی آلودگی نمونه‌ها استفاده شد. در نهایت نتایج به دست آمده در نرم‌افزار SPSS مورد آنالیز آماری قرار گرفتند. یافته‌ها: آلودگی به رنگ تارترازین تنها در سه نمونه (12%) پولکی مشاهده شد و نمونه‌های نبات عاری از این رنگ بود. غلظت رنگ در نمونه‌های مثبت حداکثر 1 میلی گرم در کیلوگرم محاسبه گردید.با درنظر گرفتن اطلاعات مصرف و میانگین وزن افراد مورد مطالعه (67 کیلوگرم) بیشینه دریافتی روزانه تارترازین از این طریق در جامعه 075/ میکروگرم به ازا هرکیلوگرم وزن بدن محاسبه شد که نسبت به دامنه بالای حد قابل قبول دریافت این رنگ (5/7 میلی گرم به ازای هر کیلوگرم وزن بدن) مقدار بسیار ناچیزی است. نتیجه‌گیری: شیوع کم تقلب استفاده از تارتارازین در کل نمونه‌ها احتمالا ناشی از تدوام کنترل این تقلب توسط سازمان‌های ناظر به خصوص معاونت غذا و داروی استان اصفهان می‌باشد. شیوع آلودگی بالاتر در نمونه‌های پولکی و دریافت روزانه دو برابری این محصول نسبت به نبات در جامعه نشان می‌دهد که پولکی سهم بیشتری در انتقال این رنگ در رژیم غذایی ایرانی دارد. خطر دریافت تارتارازین از طریق مصرف این دو آب نبات سنتی در حال حاضر اقدام جدید قانونی در مدیریت خطر را طلب نمی‌کند.

Title: مقایسه‎ی مدل‎های رگرسیون اکولوژیکی فضایی بیزی در بررسی بروز سرطان‎ سینه در ایران

http://hsr.mui.ac.ir/index.php/jhsr/article/view/2289

مقدمه: در ایران سرطان سینه با بروز استاندارد شده‎ی سنی 25/28 در رتبه‎ی اول سرطان‎ زنان قرار دارد. با توجه به روند پیر شدن جمعیت و سیر صعودی سرطان در ایران، اهمیت پیشگیری از سرطان و وجود پراکندگی سرطان‎ها در مناطق مختلف کشور، شناسایی مناطق دارای بروز بالای سرطان سینه و آگاهی از وضعیت پراکندگی و نقش مهم‌ترین عوامل خطر بالقوه‌ی این سرطان با استفاده از مدل‌های پیشرفته‌ی آماری ضروری می‎باشد. روش‌ها: در این مطالعه‌ی کاربردی اکولوژیک، جامعه مورد مطالعه، تمام بیماران مبتلا به سرطان سینه بین سال‎های 1384 تا 1387 در ایران می‎باشند. عوامل خطر: مصرف سیگار، مصرف میوه و سبزیجات، فعالیت فیزیکی، اضافه وزن و وضعیت اقتصادی-اجتماعی می‎باشد. جهت برآورد خطر نسبی سرطان سینه، از مدل‎های گاما-پوآسن، لگ‎نرمال و (BYM یا Besag,York, Mollie) استفاده شد. یافته‌ها: تعداد کل سرطان سینه ثبت شده در سال‎های1384 تا 1387 درایران 25152 مورد بوده است. از میان مدل‎های استفاده شده، مدل BYM تعدیل نشده بهترین برازش را داشت. بدون تعدیل اثر عوامل خطر، استان‎های اصفهان و یزد و تهران بیشترین خطر بروز سرطان سینه و سیستان و بلوچستان و چهارمحال و بختیاری کمترین میزان خطر ابتلا را دارند. با تعدیل عوامل خطر، استان‎های خراسان رضوی، خرم‎آباد و همدان بیشترین خطر بروز سرطان سینه و استان‎های اردبیل و کهگیلویه و بویراحمد، کمترین خطر بروز را دارند. ارتباط مستقیم معنی‌داری بین سرطان سینه و شاخص توسعه انسانی مشاهده شد. نتیجه‌گیری: با توجه به برازش بهتر مدل BYM، توصیه می‎شود در مطالعات آینده از مدل BYM استفاده شود و با توجه به معنی‎داری متغیر شاخص توسعه انسانی، در مطالعات مشابه این متغیر در نظر گرفته شود. این مطالعه نشان داد که استان‌های اصفهان، یزد و تهران دارای بیشترین خطر بروز سرطان سینه می‎باشند.

Title: عوامل مؤثر بر تعداد فرزندان زنده به دنیا آمده زنان 15- 49 ساله استان سمنان با استفاده از رگرسیون پواسن

http://hsr.mui.ac.ir/index.php/jhsr/article/view/2489

مقدمه: در مطالعات جمعیتی مقوله باروری جایگاه ویژه‌ای دارد. باروری یکی از رویدادهای طبیعی جمعیت و از عناصر مهم رشد آن می‌باشد. در تبیین عوامل مؤثر بر باروری عوامل متعدد اقتصادی، سیاسی و فرهنگی نقش دارند که محققان جمعیت‌شناسی در حوزه باروری به هر یک از این ابعاد، از جهات مختلف پرداخته‌اند. هدف از مطالعه حاضر بررسی عوامل مؤثر روی تعداد فرزندان زنده به دنیا آمده، به عنوان یکی از شاخص‌های تعیین‌کننده باروری، با استفاده از رگرسیون پواسن است. روش‌ها: 405 زن 15-49 ساله حداقل یک بار ازدواج کرده استان سمنان در سال 1391 با روش نمونه‌گیری طبقه‌بندی تصادفی انتخاب و با استفاده از پرسش‌نامه ساختار یافته بررسی شدند. به منظور مدلسازی تعداد فرزندان زنده به دنیا آمده بر روی محل تولد، سطح تحصیلی، وضعیت شغلی و نوع ازدواج زنان از مدل رگرسیون پواسن و به‌منظور برازش مدل از نرم‌افزار SPSS 17 استفاده شد. یافته‌ها: از میان چهار متغیر پیش‌بین محل تولد، سطح تحصیلی، وضعیت شغلی و نوع ازدواج، تنها دو متغیر محل تولد و وضعیت تحصیلی بر روی تعداد فرزندان به دنیا آمده تأثیر معنی‌دار داشتند (001/0>p-value). زنان متولد روستا و با تحصیلات زیردیپلم تعداد فرزندان بیشتری نسبت به زنان متولد شهر و با تحصیلات دیپلم و بالاتر داشتند. نتیجه‌گیری: از آن‌جا که متغیر تعداد فرزندان زنده به دنیا آمده، متغیری شمارشی و گسسته است، استفاده از رگرسیون پواسن برای مدلسازی آن، مدلی کاراتر از رگرسیون خطی ارائه می‌دهد.

Title: بررسی کارایی حذف نیترات با استفاده از زئولیت طبیعی کلینوپتیلولیت اصلاح شده و اصلاح نشده از محلول‌های آبی

http://hsr.mui.ac.ir/index.php/jhsr/article/view/2129

مقدمه: هدف از این مطالعه بررسی حذف نیترات با استفاده از زئولیت طبیعی کلینوپتیلولیت اصلاح شده و اصلاح نشده، ازمحلول‌های آبی مي‌باشد. روش‌ها: در این مطالعه، با توجه به راندمان پايين حذف توسط زئولیت اصلاح نشده، ازکلرید روی و فروسولفات برای اصلاح جاذب استفاده شد. پارامترهای مورد مطالعه pH (12-1)، زمان تماس (186-2 دقیقه)، غلظت نیترات (mg/l100-50) و دوز جاذب ( g/l20-5) بودند. یافته‌ها: راندمان حذف نیترات توسط زئولیت اصلاح نشده در حدود 40% بود، بنابراین در آزمایشات بعدی، زئولیت اصلاح شده به عنوان جاذب مورد استفاده قرار گرفت. شرایط مطلوب حذف نیترات برای زئولیت اصلاح شده با کلرید روی و فروسولفات در 5 و 3pH=، زمان تماس 66 و 66 دقیقه، دوز جاذب g/l 15 و 20 و غلظت اولیه نیتراتmg/l  100 و 50، به دست آمد. در این شرایط، حداکثر حذف نیترات به ترتیب 82/78٪ و 82/90٪ به دست آمد. فرآیند جذب توسط زئولیت اصلاح شده با کمک کلریدروی از ایزوترم فروندلیچ و با کمک فروسولفات از ایزوترم لانگمیر تبعیت می‌کند. نتیجه‌گیری: نتایج نشان داد که زئولیت اصلاح شده به دلیل جایگاه‌های مختلف جذب، ساده بودن سیستم و کارایی بالای حذف نسبت به زئولیت اصلاح نشده، در حذف نیترات از محلول‌های آبی کارآمدتر می‌باشد. بنابراین در غلظت‌های بالای نیترات مناسب‌ترین جاذب زئولیت اصلاح شده با کلرید روی بوده، در حالی که در غلظت‌های کم نیترات، زئولیت اصلاح شده با فروسولفات بالاترین کارایی حذف را نشان داد.

Title: بررسی کارایی صافی دانه درشتِ با جریان افقی در ارتقای کیفیت پساب خروجی از زلالسازِ لاگون‌های با هوادهی بوکان

http://hsr.mui.ac.ir/index.php/jhsr/article/view/2184

مقدمه: لزوم ارتقای کیفیت پسابِ لاگون‌ها یکی از اولویت‌های زیست محیطی طرح‌های فاضلاب است. لذا مطالعه حاضر با هدف بررسی عملکرد صافی دانه درشت با جریان افقی در بهبود پارامترهای جامدات معلق، اکسیژن مورد یاز شیمیایی، کدورت و کلیفرم مدفوعی در پساب خروجی از زلالساز لاگون‌های با هوادهی به منظور تأمین استانداردهای ملی صورت گرفت. روش‌ها: این مطالعة تجربی در مقیاس پایلوت در محل تصفیه‌خانه فاضلاب شهر بوکان انجام شد. طراحی، ساخت و نصبِ پایلوتِ صافی دانه درشت با جریان افقی بر اساس رهنمودهای وجلین صورت گرفت. عملکرد صافی در سه نرخ 5/0، 1 و 5/1 مترمکعب بر مترمربع در ساعت در حذف جامدات معلق، اکسیژن مورد نیاز شیمیایی، کدورت و کلیفرم مدفوعیِ پساب خروجی از زلالساز لاگون‌های با هوادهی مورد بررسی قرار گرفته و نتایج حاصله، با آنالیز کوواریانس (ANCOVA) به وسیله نرم‌افزار SPSS 16 مورد تحلیل قرار گرفتند. یافته‌ها: میانگین پارامترهای جامدات معلق، اکسیژن مورد نیاز شیمیایی، کدورت و کلیفرمِ مدفوعی پساب خروجی از صافی دانه درشت با جر یان افقی در نرخ‌های مختلف فیلتراسیون اختلاف معنی‌داری داشتند (001/0 P<). بر این اساس، صافی دانه درشت با جریان افقی در بارگذاری بهینة 5/0 مترمکعب بر مترمربع در ساعت، مقدار کل جامدات معلق، اکسیژن موردنیاز شیمیایی و کدورت پساب زلالساز لاگون های با هوادهی را به طور میانگین به ترتیب به mg/L 21، mg/L 35 و NTU 33/8 کاهش داد. نتیجه‌گیری: صافی دانه درشت با جریان افقی به دلیل راندمان مطلوب و راهبری آسان، می‌تواند به عنوان گزینة مناسب برای ارتقای کیفیت پسابِ زلالسازِ لاگون‌های با هوادهی جهت تأمین استاندارهای ملی مورد استفاده قرار گیرد.

Title: بررسی فعالیت فتوکاتالیستی تیتانیم دی اکسید ترکیب شده با زئولیت در حذف رنگ سافرانین در آب

http://hsr.mui.ac.ir/index.php/jhsr/article/view/2262

مقدمه: یکی از مهم‌ترین آلاینده‌های محیط زیست در پساب صنایع، رنگ می‌باشد که برای انسان خطرناک بوده و محیط زیست را آلوده می‌نماید. هدف از این پژوهش، تعیین فعالیت فتوکاتالیستی تیتانیم دی اکسید (TiO2) ترکیب شده با زئولیت در حذف رنگ سافرانین در فاز آبی بود. روش‌ها: نانوذرات TiO2 به روش سل- ژل تولید گردید و ml 5/2 سل- ژل بر روی زئولیت پوشش داده شد. رنگ سافرانین قرمز به عنوان آلاینده مدل انتخاب گردید. آزمایشات با استفاده از دستگاه میکروسکوپ الکترونی رویشی (Scanning Electron Microscopy) و دستگاه پراش پرتو X (X-ray diffraction) جهت تعیین ساختار و مورفولوژی TiO2 انجام شد. تغییرات شدت رنگ توسط دستگاه اسپکتروفتومتر در طول موج 518 نانومتر اندازه‌گیری شد. تأثیر پوشش‌دهی TiO2 بر روی زئولیت، شدت UV، غلظت اولیه رنگ و نوع بستر فتوکاتالیست در راندمان فتوکاتالیستی مورد بررسی قرار گرفت. یافته‌ها: نتایج XRD نانوذرات TiO2 دارای دو فاز آناتاز (5/80%) و روتایل (5/19%) بود و یکنواختی نانوذرات در تصاویر SEM آشکار بود. راندمان حذف TiO2 پوشش داده شده بر روی زئولیت بیشتر از فقط پوشش TiO2 بود. با افزایش شدت تابش UV از w/m2 4/2 به w/m2 9/3 سبب افزایش راندمان فتوکاتالیستی شد و زمانی که غلظت رنگ سافرانین قرمز از mg/L 10 به mg/L 50 افزایش می‌یافت راندمان از 61/91% به 91/77% کاهش یافت. بستر متحرک فتوکاتالیست TiO2/Zeolite نیز دارای راندمان بالاتری نسبت به بستر ثابت فتوکاتالیست بود. نتیجه‌گیری: فتوکاتالیست TiO2/Zeolite برای حذف رنگ سافرانین قرمز در فاز آبی بسیار مؤثرتر از فتوکاتالیست TiO2 است، به عبارت دیگر پوشش دادن TiO2 بر روی زئولیت،راندمان حذف رنگ از محیط آبی را افزایش می‌دهد. در مواردی که کدروت عمده‌ترین مانع در استفاده از فتوکاتالیست TiO2/Zeolite با بستر متحرک در تصفیه آب می‌باشد، پیشنهاد می‌شود از فتوکاتالیست با بستر ثابت، به علت عدم ایجاد کدروت در آب، استفاده شود.
